# Probiotics Improve Gastrointestinal Function and Life Quality in Pregnancy

**DOI:** 10.3390/nu13113931

**Published:** 2021-11-03

**Authors:** Albert T. Liu, Shuai Chen, Prasant Kumar Jena, Lili Sheng, Ying Hu, Yu-Jui Yvonne Wan

**Affiliations:** 1 Department of Obstetrics and Gynecology, University of California, Davis, CA 95817, USA; atliu@ucdavis.edu; 2 Division of Biostatistics, Department of Public Health Science, University of California, Davis, CA 95616, USA; shschen@ucdavis.edu; 3 Department of Pathology and Laboratory Medicine, University of California, Davis, CA 95817, USA; prasantjenacps@gmail.com (P.K.J.); fine919@163.com (L.S.); yighu@ucdavis.edu (Y.H.)

**Keywords:** GI function, intestinal motility, fecal microbiota, dysbiosis, bile acids, metabolomics, *Akkermansia muciniphila*, bile salt hydrolase

## Abstract

We studied whether probiotics were beneficial for hormonal change-associated dysbiosis, which may influence the enteric nervous system and GI function during early pregnancy. The study was 16 days consisting of two cycles of six daily probiotics mainly *Lactobacillus* and 2 days without probiotics. Daily surveys were conducted to monitor GI function and life quality. A subset of the participants who contributed fecal specimens was used for microbiota metagenomic sequencing, metabolomics, and quantification of bacterial genes to understand potential underlying mechanisms. Statistical analyses were done by generalized linear mixed-effects models. Thirty-two obstetric patients and 535 daily observations were included. The data revealed that probiotic supplementation significantly reduced the severity of nausea, vomiting, constipation, and improved life quality. Moreover, a low copy number of fecal *bsh* (bile salt hydrolase), which generates free bile acids, was associated with high vomiting scores and probiotic intake increased fecal *bsh*. In exploratory analysis without adjusting for multiplicity, a low fecal α-tocopherol, as well as a high abundance of *Akkemansia muciniphila*, was associated with high vomiting scores and times, respectively. The potential implications of these biomarkers in pregnancy and GI function are discussed. Probiotics likely produce free bile acids to facilitate intestinal mobility and metabolism.

## 1. Introduction

Nausea and vomiting affect about 85% of pregnant women and have a significantly negative impact on life quality during early pregnancy. Vitamin B_6_, antihistamine doxylamine, and metoclopramide may benefit patients who have nausea and vomiting during pregnancy. Alternatively, holistic remedies may be useful. Overall, there is a lack of strong evidence that any of these options can effectively relieve nausea and vomiting during pregnancy [1,2].

Pregnancy-associated nausea, constipation, stomach upset, bloating, fatigue, etc. have no apparent structural abnormality, hence are referred to as functional gastrointestinal (GI) disorders. Elevated estrogen and progesterone during pregnancy change gut microbiome composition and function, which can significantly impact GI function [3,4]. Whether probiotics are an option to reduce GI dysfunction and emotional distress, which are regulated by the enteric nervous system, has never been studied. 

Gut microbiota via generated metabolites affect host health. Among these metabolites, bile acids are the obvious links that explain how gut microbes affect GI health because bile acids are jointly produced by the liver and gut microbial enzymes. Moreover, gut dysbiosis is accompanied by dysregulated bile acid synthesis [5,6]. In addition, through a G protein-coupled bile acid receptor (TGR5), Mas-related G-protein coupled receptor member X4 (MRGPRX4), and farnesoid X receptor (FXR), bile acids regulate smooth muscle contraction, defecation, and sensation in addition to lipid metabolism and absorption [7,8,9,10]. Thus, we tested a hypothesis that probiotics may shift gut microbiota and metabolites to affect GI function during early pregnancy. 

We analyzed whether probiotic supplementation influenced GI dysfunction and life quality within 24 h through daily surveys. Fecal specimens were collected from a subset of participants. Based on 32 enrolled participants and observations over hundreds of days, data showed that probiotics significantly improved daily nausea and vomiting scores as well as overall life quality. In addition, fecal biomarkers including vitamin E, *A. muciniphila*, and the copy number of bile salt hydrogenase gene (*bsh,* BSH) may predict the severity of vomiting. Furthermore, probiotic intake increased the abundance of BSH-generating bacteria, which produced free bile acids thereby enhancing GI motility and metabolism leading to improved GI function.

## 2. Materials and Methods

### 2.1. Study Design

The study protocol was approved by the University of California, Davis Institutional Review Board. The used probiotics are nutritional supplements. Because the intention was not to treat a disease, the study received a waiver for the Investigational New Drug Application from the US Food and Drug Administration. 

It is challenging to compare the commensal microbiome among people due to its variability. Thus, we performed a study comparing the effect of probiotics within a participant over 24 h with or without taking probiotics. The duration of the study was 16 days consisting of 2 cycles of 6 daily probiotics and 2 days without probiotics. Participants entered the study without taking probiotics (Day 0) followed by taking probiotics (Probiotics 10, 2 capsules/day Nature’s Bounty, Ronkonkoma, NY) for 6 days and off probiotics for 2 days. Then, the same cycle was repeated. Each capsule contained inulin (200 mg) and 10 probiotics including *L. plantarum 299v*, *L. bulgaricus Lb-87*, *L. paracasei DSM 13434*, *L. plantarum DSM 15312*, *L. salivarius Ls-33*, *L. brevis Lbr-35*, *L. acidophilus La-14*, *B. lactis Bl-04*, *L. paracasei Lpc-37*, and *L. casei Lc-11* (10 billion live cultures at the time of manufacture). 

The inclusion and exclusion criteria are described in Supplemental Materials. A sample size of 32 patients (each with 17 daily observations, including Day 0) has 87% power to detect a change of 20% of 24-h nausea times by taking probiotics, with a significance level of 0.05. This is based on a Poisson mixed-effects model using 2000 bootstrap replicates generated from 11 preliminary patients. 

### 2.2. Data and Specimen Collection

The participants were asked to enter a daily survey to understand whether probiotic intake influenced GI function within 24 h. The survey questions included daily nausea and vomiting times as well as 17 questions that evaluated life quality. Scores ranged from 1-5: score 1, none; 2, 1-2 times/day (not too bad or fair); 3, 3 times/day (bad); 4, 4-5 times/day (awful); 5, all the time (cannot function). In addition, information regarding constipation and daily nausea hours was obtained for a subset of patients. 

Fecal specimens were collected using an OMNIgene•GUT kit (Ontario, Canada). The preference was 1 specimen before taking probiotics, 2 without taking probiotics, and 3 while taking probiotics in the past 24 h. The goal was to collect 6 specimens per participant. Gut metabolites and microbiota were measured in a pre-determined subset of 14 enrollees. Due to unavailable/unmeasurable samples, gut metabolites and microbiota were quantified in 11 of the 14 enrollees (79%), and bacterial genes in 25 of all 32 patients (78%).

### 2.3. Quantification of Bacteria Genes

Fecal DNA was extracted using a ZR Fecal DNA MiniPrep Kit (Zymo Research, Irvine, CA, USA), quantified by NanoDrop (Thermo Fisher Scientific, West Sacramento, CA, USA), and amplified using primer sequences of butyryl-coenzyme-A-CoA transferase (*bcoA*) and butyrate kinase (*buk*), which generate butyrate as well as bile acid inducible 7α-dehydroxylating operon (*baij*) and bile salt hydrolase (*bsh*)*,* which produces bile acids (Table S1). These genes were selected because the used probiotics contain inulin, which supports the growth of butyrate-generating bacteria, and because the used probiotics also contain BSH-producing bacteria. Bacterial DNA concentration was calculated using standard curves of diluted synthetic DNA fragments. 

### 2.4. Shotgun Metagenomic Sequencing

DNA samples were converted to libraries using a NuGEN Celero Library Preparation Kit (NuGEN/Tecan, Redwood City, CA, USA) and NuGEN dual-indexed sequencing adapters. The libraries were amplified by PCR, analyzed via microcapillary gel electrophoresis, and combined in a pool at equimolar ratios. The pool was quantified with a Kapa Library Quant kit (Kapa Biosystems/Roche, Basel, Switzerland) on a QuantStudio 5 real-time PCR system (Applied Biosystems, Foster City, CA, USA) and sequenced on an Illumina HiSeq4000 lane (Illumina, San Diego, CA, USA) with paired-end 150 bp reads.

### 2.5. Bioinformatic Analysis

The raw read data was filtered using HTStream (version 1.0.0) that included screening for contaminants, removal of duplicated reads, quality-based trimming, and adapter trimming [11]. Metagenomic classification of the processed reads was accomplished using Kraken2 (version 2.0.8-beta) [12]. Bracken (version 2.1) was then used to estimate abundance at each taxonomic level across all the classified organisms [13]. To quantify the diversity of microbiomes, Shannon’s and Simpson’s diversity indices were calculated for each taxonomic level. 

### 2.6. Untargeted Metabolomic Analysis

Fecal metabolite levels were quantified by gas chromatography time-of-flight mass spectrometry (GC-TOFMS). Acquired spectra were processed using the BinBase database, filtered, and matched with the Mass Spectral Library of 1200 metabolite spectra with retention index and mass spectrum information. 

### 2.7. Statistical Analysis

Overview of statistical analysis. Although the performed study was not intended to treat a disease, we used standard terminology, intention-to-treat (ITT) analysis (where the probiotic status (on/off) was based on the initial study design, and not on the actual probiotic status that the participants received) and per-protocol (PP) analysis (which only included participants completing the study without serious violations of protocol). The ITT population includes 32 participants who might not have complied with the protocols. Primary analysis was conducted in the ITT population to evaluate the effects of probiotics, which is generally a more conservative approach than PP analysis. PP analysis was also conducted as a sensitivity analysis. The co-primary outcomes were nausea and vomit times/scores, and secondary outcomes included other life quality scores and constipation. Demographics of participants were summarized using descriptive statistics. Generalized linear mixed models were used for data that was collected repeatedly for an individual (across time) [14]. Tests were two-sided with α = 0.05, except for the exploratory analysis for metabolites and microbiota abundance, where Benjamini–Hochberg procedures were used to adjust for multiplicity. Due to the exploratory nature, the adjustment for multiplicity was not required [15] but was done. Further studies to confirm the effect of probiotics on these biomarkers will be needed with an increased sample size in the future. All analyses were conducted in R 3.6.1 (R Foundation for Statistical Computing, Vienna, Austria) and SAS 9.4. (SAS Institute Inc., Cary, NC, USA). See detailed analysis in Supplemental Material.

Evaluating effects of probiotics. The effects of probiotics in the following 24 h were evaluated using ordinal logistic mixed-effects models for ordinal outcomes, Poisson mixed-effects models for count outcomes, logistic mixed-effects models for binary outcomes, and linear mixed-effects models for continuous outcomes. All models were adjusted by gestational age. Patient-specific random effects were included in the model to account for within-patient correlation. Log-transformations were employed for biomarkers if needed for normality, and fold changes (exponentiated coefficients) were reported consequently.

Exploring association between biomarkers and nausea/vomit. We further explored whether patients with high (or low) biomarker levels had more severe nausea and vomiting, and whether the effects of probiotics were heterogeneous in patients with different baseline biomarker levels. To protect family wise error rate, we only examined the 4 biomarkers which were altered by probiotic intake with raw *p*-values < 0.05 in both ITT and PP analyses. The fixed effects included probiotic status, gestational age, each biomarker of interest on Day 0, and its interaction with probiotic status. The interactions between probiotics and biomarkers on Day 0 were removed from final models due to no significance. 

Handling missing data. Gut metabolites, microbiota, constipation status, and nausea hours were measured among pre-determined subsets of participants. For example, constipation status and nausea hours were planned to be measured among the last 15 participants, and gut metabolites and species of microbiota were planned to be measured among 14 participants in the early phase, which were likely to be missing-completely at-random (MCAR). An additional 22% of participants had unavailable fecal data because fecal specimens were optional. A few participants had missing daily survey data for several days (about 2%). Missing values were handled using likelihood-based approaches in mixed-effects models, which used all available data in the model estimation and provided unbiased estimates under a missing-at-random (MAR) assumption. 

## 3. Results

### 3.1. The Effect of Probiotics on Symptoms

Table 1. Ages, BMI, gestation ages, and information related to nausea, vomiting, and constipation on day 0 for the ITT population.

Table 2 summarizes the effects of probiotics on 24-h outcomes. Probiotic intake significantly reduced all outcomes quantifying nausea and vomiting, by reducing nausea hours by 16%, nausea times by 16%, and vomit times by 33%. Probiotic intake also significantly improved 15 out of 17 secondary life quality scores. Additionally, probiotics significantly reduced constipation, defined as either stool was hard, or difficult to pass, or not all passed. Specifically, probiotics reduced hard stool but did not alter the number of bowel movements (Table 2). The above-mentioned findings remained the same with generally slightly larger effects in the PP analysis (Table S2). 

### 3.2. The Effect of Probiotics on Fecal Metabolites

There were 123 known metabolites detected. Probiotic intake increased α-tocopherol and fucose with raw *p*-values < 0.05 in the ITT population. In the PP population, the increase of α-tocopherol remained based on the raw *p* value. However, after adjusting for multiplicity, no metabolites reached a significant difference (Table S3) although the adjustment for multiplicity was not required for such exploratory analysis [15]. The result indicates that it is worth conducting further studies to confirm the effect of probiotics on α-tocopherol although the significance did not remain after the multiplicity adjustment due to the small sample size of this study.

### 3.3. The Effect of Probiotics on Fecal Microbiota

No significant effect of probiotics was noted at phylum and family levels. Shannon and Simpson diversity indexes were not significantly different at all levels either. In response to probiotic intake, the abundance of genus *Akkermansia,* as well as *A. muciniphila,* consistently decreased with raw *p* values < 0.05 in both the ITT and PP analyses, but there was no significance after adjusting for multiplicity (Table S3). The result indicates that it is worth conducting further studies to confirm the effect of probiotics on *Akkermansia* as well as *A. muciniphila* although the significance did not remain after the multiplicity adjustment due to the small sample size of this study. 

### 3.4. The Effect of Probiotics on the Butyric Acid and Bile Acid-Generating Bacteria

We took a different approach and quantified the copy number of bacterial genes. The choices were genes responsible for producing short-chain fatty acid butyrate (*bcoA*, *buk*) and bile acids (*baij*, *bsh*), which all had a significant impact on GI health. Among the studied genes, the copy number of *bsh* increased significantly by 5.41-fold due to probiotic intake (Table 3). The other three genes were not significantly altered by probiotic intake. The PP effect of probiotics had a 6.10-fold increase in *bsh* (Table S4).

### 3.5. The Relationships between Biomarkers and Symptoms

We further explored whether patients with high (or low) levels of biomarkers had more severe symptoms. Patients with high fecal α-tocopherol levels on Day 0 were significantly associated with low vomiting scores during the entire study, and low vomiting times with significance near the edge (Table S5). Additionally, high *Akkermansia* and *A. muciniphila* abundances on Day 0 were significantly associated with high vomiting times during the entire study. 

Moreover, patients with high copy numbers of *bsh* on Day 0 were significantly associated with low daily vomiting scores and low daily vomiting times during the entire study (Table 4). PP analysis reported similar effect sizes, with a significant association with low vomiting times remaining but the significance for vomiting scores was near the edge (Table S6). 

## 4. Discussion

This novel study has revealed the beneficial effects of probiotics in reducing GI dysfunction during pregnancy. It has been suggested that increased progesterone during pregnancy leads to alterations of GI motility, which may contribute to nausea and vomiting [16]. However, sex hormones affect the composition of the gut microbiome [4,17]. Thus, the dramatic changes of sex hormones during early pregnancy can alter the structure of gut microbiota thereby likely contributing to nausea, vomiting, and constipation during pregnancy. Our data supports this scenario since probiotics markedly reduced the severity of GI dysfunction. 

The enteric nervous system has 100 million neurons to secrete neuropeptides found in the central nervous system [18]. Neuropeptides have an apparent impact on host stress, they also have anti-microbial activity, therefore, affecting gut microbiota structure. Furthermore, microbiota and their regulated signaling via the gut-brain axis also affect anxiety and depression [19]. Our data showed that probiotics improved overall life quality during early pregnancy. 

Although the identified metabolites and bacteria did not show significant changes due to probiotic intake based on the adjusted *p* values, they are still interesting and worth our attention. Based on the raw *p*-value, α-tocopherol (vitamin E) increased after probiotic intake. Strikingly, high fecal α-tocopherol levels were significantly associated with low vomiting scores suggesting its predictive value. It is interesting to note that vitamin E levels, but not vitamin A levels, continuously rose with an increase in gestational age throughout pregnancy suggesting the essentialness of vitamin E for pregnancy [20]. Whether increased vitamin E as gestation age advances relieves GI dysfunction is an interesting topic. The role of vitamin E in regulating GI function remains to be explored. 

The abundance of *Akkermansia* and *A. muciniphila* was significantly associated with high vomiting times. Moreover, probiotic intake significantly reduced vomiting and the abundance of *Akkermansia* and *A. muciniphila* based on raw *p* values. *A. muciniphila* are mucin-degrading organisms that use mucus as an energy source [21]. Although mucus is integral to gut health, its level needs to be controlled by propelling to the distal GI tract. Patients who do not have normal GI motility might have excessive mucus that requires increased *A. muciniphila* to digest it. Together, a high abundance of *A. muciniphila* can be a biomarker to predict vomiting in pregnancy. 

Our early studies revealed the anti-inflammatory and metabolic effects of butyrate and its prebiotic inulin [22,23]. However, the provided probiotics, which contained inulin, did not change the abundance of butyric acid-generating bacteria. Thus, it is unlikely inulin or butyric acid had beneficial effects in improving GI motility during pregnancy. 

Remarkably, fecal *bsh* had a 5- to 6-fold increase in response to probiotic intake. Additionally, high *bsh* levels were significantly associated with low daily vomiting scores and vomiting times. However, such a relationship was not noted for the *baij* encoding bile acid 7α-dehydroxylase that converts primary bile acids into secondary bile acids. Elevated secondary bile acids are genotoxic and cause cancer [24]. In contrast, *bsh* encoded bile salt hydrolase deconjugates bile acids and is present in *Lactobacillus* and *Bifidobacterium,* which are routinely used as probiotics [25]. 

Our data showed that the provided probiotics contained 720,000 copy numbers of *bsh* per ng of DNA (not shown). By reducing bile toxicity and generating free bile acids, the enzymatic action of BSH likely increased the activity of bile acid receptors. It is known that through TGR5, and MRGPRX4, bile acids regulate intestinal motility, muscle contraction, and sensation [7,10]. The function of bile acids in the enteric nervous system warrants further investigation. Together, a low copy number of *bsh* predicts vomiting during pregnancy. Moreover, increased *bsh* reflects probiotic intake. Therefore, regulating bile acid signaling pathways may explain the benefits of probiotics in regulating GI function during early pregnancy. 

Due to the limited sample size, some of the results are exploratory. The adjustment for multiplicity was not required for the biomarkers due to the exploratory nature [15] of this study, although we further performed multiplicity adjustments. Hence, an additional study with an increased sample size is needed to confirm these exploratory findings. Other limitations of this study include not using a randomized trial and not blinding participants with a placebo. In addition, 24-h effects of post probiotic intake were evaluated, assuming no carryover effect. Hence, long-term effects of probiotics were not assessed, which might lead to potentially underestimated probiotic effects. Overall, our findings provide a basis for further investigation of the benefits of probiotics as well as bile acid singling during pregnancy.

## Figures and Tables

**Table 1:** Demographics and characteristics of the participants at enrollment (*n* = 32).

Characteristics	*n* (%) or Mean ± SD
Non-Hispanic White	
No	19 (59.4%)
Yes	13 (40.6%)
Education	
High school diploma or equivalent	1 (3.1%)
Some college	3 (9.4%)
College degree or higher	28 (87.5%)
Age	31.6 ± 3.9 years
BMI	27.5 ± 6.4
Gestational age	72.3 ± 15.6 days
Constipation ^a, b^	
No	2 (13.3%)
Yes	13 (86.7%)
Nausea times per day	7.0 ± 5.5
Nausea hours per day ^b^	10.3 ± 6.6
Nausea score (1–5)	3.6 ± 0.9
Vomiting times ^c^	2.0 ± 1.3
Vomiting score (1–5) ^c^	2.8 ± 1.0

^a^ Constipation is defined as yes to any one of the questions listed below: Stools are hard, difficult to pass, or not all passed. ^b^ Constipation status and nausea hours were measured in 15 enrolled patients. ^c^ Only participants who had vomiting during enrollment were included (*n* = 12).

**Table 2:** Estimated ITT effects of probiotics based on generalized linear mixed-effects models adjusted by gestational age.

Symptoms	Number of Participants (Observation Days)	Incidence Rate Ratio ^a^ (95% CI)	*p*-Value	Odds Ratio ^b^ (95% CI)	*p*-Value
**Nausea and Vomiting:**					
Daily nausea score (1–5)	32 (535)			0.46 (0.31, 0.67) **	<0.001
Daily vomit score (1–5) ^c^	12 (187)			0.31 (0.15, 0.65) **	0.002
Nausea hours per day	15 (255)				
Daily nausea times	32 (534)	0.84 (0.75, 0.95) **	0.005		
Daily vomiting times ^c^	12 (200)	0.84 (0.77, 0.92) **	<0.001		
Nausea and Vomiting:	32 (535)	0.67 (0.50, 0.90) **	0.008		
**Mood/Life Quality:**					
(1) Fatigue (1–5)	32 (534)			0.41 (0.27, 0.61) **	<0.001
(2) Emotional (1–5)	32 (534)			0.70 (0.45, 1.07)	0.10
(3) Dry heaves (1–5)	32 (532)			0.47 (0.30, 0.75) **	0.001
(4) Worse when exposed to certain smells (1–5)	32 (535)			0.37 (0.25, 0.57) **	<0.001
(5) Feeling blue (1–5)	32 (535)			0.59 (0.36, 0.97) *	0.04
(6) Poor appetite (1–5)	32 (533)			0.42 (0.28, 0.63) **	<0.001
(7) Worse when exposed to certain foods (1–5)	32 (535)			0.44 (0.28, 0.67) **	<0.001
(8) Worn-out (1–5)	32 (535)			0.46 (0.31, 0.69) **	<0.001
(9) Fed up with being sick (1–5)	32 (535)			0.50 (0.33, 0.76) **	0.001
(10) Frustrated in response to statement that your symptoms are part of normal pregnancy (1–5)	32 (535)			0.68 (0.42, 1.08)	0.10
(11) Moody (1–5)	32 (535)			0.60 (0.38, 0.93) *	0.02
(12) Everything is an effort (1–5)	32 (535)			0.43 (0.28, 0.65) **	<0.001
(13) Took longer to get things done than usual (1–5)	32 (535)			0.38 (0.25, 0.57) **	<0.001
(14) Difficulty maintaining normal social activities (1–5)	32 (535)			0.42 (0.27, 0.65) **	<0.001
(15) Difficulty shopping for food (1–5)	32 (534)			0.43 (0.27, 0.67) **	<0.001
(16) Difficulty preparing meals (1–5)	32 (535)			0.29 (0.18, 0.46) **	<0.001
(17) Cut down on the time at work or other activities (1–5)	32 (533)			0.42 (0.27, 0.66) **	<0.001
**Constipation:**					
Bowel movement (yes/no)	15 (255)			0.93 (0.45, 1.93)	0.85
Number of Bowel movement	15 (255)	0.94 (0.73, 1.20)	0.605		
Stools are difficult to pass (yes/no)	15 (255)			0.54 (0.26, 1.12)	0.10
Stools are hard (yes/no)	15 (255)			0.23 (0.10, 0.50) **	<0.001
Not all stools passed (yes/no)	15 (254)			0.47 (0.22, 1.02)	0.06
Constipation (yes/no) ^d^	15 (255)			0.37 (0.18, 0.79) *	0.010

^a^ Incidence rate ratio (IRR) (= mean probiotics / mean no probiotics) from Poisson mixed-effects model for count symptoms. IRR < 1 means that probiotics reduce symptoms. ^b^ Odds ratios on increasing to the next higher level of symptom with probiotics, from ordinal logistic mixed-effects model for ordinal outcomes. The symptoms are on a 5-point Likert scale, with 1 = lowest and 5 = highest. OR < 1 means that probiotics reduce the score. ^c^ Participants without vomiting at enrollment were excluded from analysis. ^d^ Constipation is defined as yes to any one of the questions listed below: Stools are hard, difficult to pass, or not all passed. ** indicates *p* < 0.01, * indicates *p* < 0.05.

**Table 3:** Estimated ITT effects of probiotics on gene copy numbers, based on linear mixed-effects models adjusted by gestational age. ** indicates *p* < 0.01.

Genes	Number of Participants (Observation Days)	Fold Change ^a^ (95% CI)	*p*-Value
**Butyric acid-producing genes**			
*bcoA*	25 (155)	0.94 (0.74, 1.18)	0.57
*buk*	25 (155)	0.96 (0.71, 1.31)	0.81
**Bile acid-producing genes**			
*bsh*	25 (155)	5.41 (3.13, 9.34) **	<0.001
*baiJ*	25 (155)	0.79 (0.49, 1.26)	0.31

^a^ Gene copy numbers were log-transformed, and fold changes (exponentiated coefficients) due to probiotic intake were reported. Fold change > 1 means that the gene copy number increases after probiotic intake. *bcoA*: butyryl-coenzyme-A-CoA transferase; *buk*: butyrate kinase; *baiJ*: bile acid inducible 7α-dehydroxylating operon; *bsh*: bile salt hydrolase.

**Table 4:** Association between *bsh* copy number on Day 0 and nausea and vomit in ITT population, based on generalized linear mixed-effects models. Fixed effects included probiotics, gestational age, *bsh* at Day 0, and its interaction between probiotics. Interaction between *bsh* and probiotics was removed from the final model due to no significance. ** indicates *p* < 0.01, * indicates *p* < 0.05.

	Biomarker = Log-Transformed Bsh Copy Number				
Symptoms	Number of Participants (Observation Days)	Incidence Rate Ratio ^a^ (95% CI)	*p*-Value	Odds Ratio ^b^ (95% CI)	*p*-Value
Daily nausea score (1–5)	25 (416)			0.71 (0.34, 1.48)	0.36
Daily vomit score (1–5) ^b^	10 (166)			0.20 (0.06, 0.72) *	0.02
Daily nausea times	25 (415)	0.94 (0.75, 1.18)	0.60		
Daily vomiting times ^c^	10 (166)	0.50 (0.31, 0.81) **	0.005		

^a^ Incidence rate ratio (IRR) of a 1-unit increase in log-transformed *bsh* on Day 0, based on Poisson mixed-effects model for count outcomes. IRR < 1 means that patients with high *bsh* copy numbers on Day 0 are associated with a low outcome during the entire study (after adjusting for probiotic effect). ^b^ Odds ratios on increasing to the next higher level of symptoms when there is a 1-unit increase in log-transformed *bsh* on Day 0, based on an ordinal logistic mixed-effects model for ordinal symptoms. The symptoms are on a 5-point Likert scale, with 1 = lowest and 5 = highest. OR < 1 means that patients with a high *bsh* level on Day 0 are associated with low symptom scores during the entire study (after adjusting for probiotic effect). ^c^ Participants without vomiting at enrollment were excluded.

## Data Availability

The data presented in this study are available on request from the corresponding author.

## Supplementary Materials

The following are available online at https://www.mdpi.com/article/10.3390/nu13113931/s1, Supplemental Materials: Supplemental Methods, Table S1: Primer used for amplification of bacterial genes, Table S2: Estimated PP effects of probiotics, based on generalized linear mixed-effects models adjusted by gestational age, Table S3: Estimated ITT and PP effects of probiotics on bacteria and metabolites based on linear mixed-effects models adjusted by gestational age, Table S4: Sensitivity analyses in PP population, Table S5: Association between biomarkers on Day 0 and symptoms in ITT and PP population, based on generalized linear mixed-effects models, and Table S6: Sensitivity analyses in PP population.
