# Supplementary material for: Probiotics Improve Gastrointestinal Function and Life Quality in Pregnancy
Source: Nutrients. 2021 Nov 3;13(11):3931. doi: 10.3390/nu13113931 (PMC8624890; doi:10.3390/nu13113931)
Supplement: Supplementary file 1 [file nutrients-13-03931-s001.zip › nutrients-1433141-supplementary.pdf]

SUPPLEMENTARY APPENDIX:

Table of Contents:

Page 1: Table of Contents

Page 2-3: Supplemental Materials

Page 4: Table S1

Page 5-6: Table S2

Page 7-8: Table S3

Page 9: Table S4

Page 10-11: Table S5

Page 12: Table S6

Page 13: Appendix References

Investigators:

Albert T. Liu M.D.[1]; Shuai Chen Ph.D.[2]; Prasant Kumar Jena Ph.D.[3]; Lili Sheng Ph.D.[3]; Ying Hu Ph.D.[3]; Yu-Jui Yvonne Wan Ph.D.[3]

[1] Department of Obstetrics and Gynecology
[2] Division of Biostatistics, Department of Public Health Science
[3] Department of Pathology and Laboratory Medicine, University of California, Davis, Sacramento, CA 95817, USA

SUPPLEMENTAL MATERIALS:

Supplemental Methods:

Inclusion and Exclusion Criteria:

Inclusion criteria consisted of: (1) First trimester healthy or low-risk pregnant women who reported GI symptoms such as nausea, vomiting, and constipation. The definition of healthy or low-risk pregnant women had systolic and diastolic blood pressure < 140 mmHg and < 90 mmHg, respectively, and hemoglobin ≥ 10.0 g/dL. They were HIV-negative and had no symptoms of kidney or urinary tract infection. (2) They were 14 weeks gestation or less with a confirmed fetal heartbeat. (3) The pregnant women were 18 years of age or older.

Exclusion criteria were: (1) High-risk pregnant women with existing health conditions such as high blood pressure, Type 1 diabetes, HIV-positive, or were infected with Hepatitis B, Syphilis, or other sexually transmitted diseases; (2) multiple gestations or a history of recurrent miscarriages (more than two consecutive miscarriages); (3) prior bariatric surgery or conceived through an IVF program; (4) serious weight loss (more than 5 pounds) during pregnancy; (5) history of irritable bowel syndrome or other non-pregnancy related GI dysfunction; (6) taken prescription medicines including antibiotics in the past one month. In addition, patients who had hyperemesis gravidarum were excluded because the intention was not to treat.

Details of analysis population, statistical model building, and interpretation:

Analysis Population. The primary analysis was conducted in the intention-to-treat analysis (ITT) population, including a total of 32 participants with 535 daily observations, where the probiotics status (on/off) is based on the initial study design, and not on the actual probiotics status that the participants received. Additional sensitivity analyses were conducted in the per-protocol (PP) population, defined as a subset of the ITT population who completed the study without strong violations such as not taking probiotics. Participants with mild violations (e.g., reducing probiotic dosage) were included in the PP population. Seven participants did not stop taking probiotics after day 14, and their 24-hour daily observations after that were excluded. This led to 27 participants with 450 daily observations in the PP population. Twelve participants with vomiting time ≥1 and vomiting score >1 at enrollment (day 0) were included for vomiting analysis.

Evaluating effects of probiotics on symptoms. All generalized linear mixed-effects models include fixed effects of probiotic status (on/off, based on the study design even the patient violated) and linear gestational age at measurement. The quadratic gestational age effect was also considered but removed in final models due to no significance. A random intercept was included in the model to account for within-patient correlation. A random slope for gestational age was added to the models if significant in the likelihood ratio test. The effects of probiotics on 24-hours were scored with a 5-point Likert scale (nausea and vomiting scores, and other secondary life quality scores) and evaluated using ordinal logistic mixed-effects models with cumulative logit link, and odds ratios (OR) on increasing to the next higher level score with probiotics were reported, where OR<1 means that probiotics reduced the score. The effects of probiotics on 24-hour count outcomes (nausea and vomiting times, nausea hours, number of bowel movements) were evaluated using Poisson mixed-effects models with log link, and incidence rate ratio (IRR = $mean_{probiotics}$ / $mean_{no\ probiotics}$) were reported, where IRR<1 means that probiotics reduced the mean outcome. For example, IRR=0.8 means that probiotics reduced the mean outcome by 1-0.8 = 20%. The effects of probiotics on constipation symptoms (yes/no) were evaluated using logistic mixed-effects models for a binary outcome, where OR<1 means that probiotics improved constipation.

Evaluating effects of probiotics on biomarkers. The effects of probiotics on biomarkers in the next day's fecal samples (bacterial gene copy numbers, metabolomics,

gut microbiome abundance, Shannon's and Simpson's Indices for each level) were evaluated using linear mixed-effects models. All models include fixed effects for probiotic status (on/off, based on the study design even if the patient violated) and linear gestational age at measurement. A random intercept was included in the model to account for within-patient correlation. For gut microbiome abundance and Shannon/Simpson's Index, the estimated differences (=$mean_{probiotics} - mean_{no\ probiotics}$) were reported, where difference<0 means that probiotics reduced the outcome. Bacterial gene copy numbers and metabolomics were log-transformed, and hence fold changes (exponentiated coefficients) at probiotics with respect to no probiotics were reported. Fold change>1 means that the outcome increased with probiotics. Benjamini-Hochberg procedures were used to adjust raw *p*-values for 123 metabolites as well as top 5 phylum, 15 families, 50 genus, and 100 species. Based on the results, we identified 4 biomarkers of interest, which were affected by probiotics in both ITT and PP analyses consistently with raw *p*-values<0.05. For the purpose of exploration, we identified those biomarkers with raw *p*-values<0.05 even if they are not significant after adjusting for multiplicity.

Exploring association between biomarkers and nausea/vomit. We further explored whether the 4 biomarkers of interest can predict nausea and vomit and whether the effects of probiotics are heterogeneous in patients with different baseline biomarker levels, by performing a secondary set of models. Per the guidance of the FDA, since the biomarkers are potentially affected by probiotics, the post-baseline biomarkers should not be adjusted in the models [1]. For each outcome, four separate models were fitted for each of the four biomarkers. The fixed effects included probiotic status, gestational age, each biomarker of interest on Day 0, and its interaction with probiotic status. A random intercept was included in the model to account for within-patient correlation. To protect family wise error rate, we only examined 4 biomarkers of interest. The interactions between baseline biomarkers and probiotics were removed from final models due to no significance, indicating that: (1) no significant heterogeneity in effects of probiotics was observed among patients with different biomarker levels; (2) for two patients with a 1-unit difference in biomarker levels at Day 0, their expected difference in nausea/vomit (log- or logit- scaled, represented by IRR or OR) are constant if both are taking probiotics or not, as reported in Table 4, S5, and S6. For example, IRR = 0.6 for baseline log-*bsh* (on Day 0) means that, if Patient A is 1-unit more than Patient B in log-*bsh* level at Day 0, then Patient A is expected to have 40% (=1-0.6) less vomit (or nausea) times than Patient B during the entire study, where the rate 40% is constant if both patients are on or off probiotics.

Supplemental Tables:

Table S1. Primer used for amplification of bacterial genes

**Gene name**	**5'-3' sequence**	**Reference**
baiJ	F: TCAGGACGTGGAGGCGATCCA<br>R: TACRTGATACTGGTAGCTCCA	[2]
bsh	F: ATGGGCGGACTAGGATTACC<br>R: TGCCACTCTCTGTCTGCATC	[3]
bcoA	F: GCIGAICATTTCACITGGAAYWSITGGCAYATG<br>R: CCTGCCTTTGCAATRTCIACRAANGC	[4]
buk	F: TGCTGTWGTTGGWAGAGGYGGA<br>R: GCAACIGCYTTTTGATTTAATGCATGG	[5]

Table S2. Estimated PP effects of probiotics, based on generalized linear mixed-effects models adjusted by gestational age. ** indicates *p* < 0.01, * indicates *p* < 0.05.

Symptoms	Number of Participants (Observation Days)	Incidence Rate Ratio [a] (95% CI)	*p*-Value	Odds Ratio [b] (95% CI)	*p*-Value
**Nausea and Vomiting:**					
Daily nausea score (1-5)	27 (450)			0.42 (0.27, 0.64) **	<0.001
Daily vomit score (1-5) [c]	11 (182)			0.30 (0.14, 0.63) **	0.002
Nausea hours per day	13 (216)	0.81 (0.71, 0.92) **	0.001		
Daily nausea times	27 (449)	0.80 (0.73, 0.89) **	<0.001		
Daily vomiting times [c]	11 (186)	0.67 (0.50, 0.91) *	0.011		
**Mood/Life Quality:**					
1) Fatigue (1-5)	32 (534)			0.39 (0.25, 0.61) **	<0.001
2) Emotional (1-5)	32 (534)			0.78 (0.49, 1.24)	0.301
3) Dry heaves (1-5)	32 (532)			0.45 (0.27, 0.75) **	0.002
4) Worse when exposed to certain smells (1-5)	32 (535)			0.38 (0.24, 0.60) **	<0.001
5) Feeling blue (1-5)	32 (535)			0.56 (0.31, 1.01)	0.053
6) Poor appetite (1-5)	32 (533)			0.37 (0.24, 0.58) **	<0.001
7) Worse when exposed to certain foods (1-5)	32 (535)			0.43 (0.26, 0.69) **	0.001
8) Worn-out (1-5)	32 (535)			0.47 (0.30, 0.73) **	0.001
9) Fed up with being sick (1-5)	32 (535)			0.35 (0.22, 0.57) **	<0.001
10) Frustrated in response to statement that your symptoms are part of normal pregnancy (1-5)	32 (535)			0.63 (0.38, 1.06)	0.082
11) Cannot enjoy pregnancy (1-5)	32 (535)			0.61 (0.38, 1.00) *	0.050
12) Everything is an effort (1-5)	32 (535)			0.36 (0.23, 0.57) **	<0.001
13) Took longer to get things done than usual (1-5)	32 (535)			0.28 (0.17, 0.44) **	<0.001

14) Difficulty maintaining normal social activities (1-5)	32 (535)			0.42 (0.26, 0.67) **	<0.001
15) Difficulty shopping for food (1-5)	32 (534)			0.34 (0.20, 0.57) **	<0.001
16) Difficulty preparing meals (1-5)	32 (535)			0.24 (0.14, 0.41) **	<0.001
17) Cut down on the time at work or other activities (1-5)	32 (533)			0.42 (0.26, 0.67) **	<0.001
**Constipation:**					
Bowel movement (yes/no)	13 (216)			0.84 (0.37, 1.91)	0.672
Number of Bowel movement	13 (216)	0.95 (0.72, 1.25)	0.719		
Stools are difficult to pass (yes/no)	13 (216)			0.76 (0.32, 1.82)	0.536
Stools are hard (yes/no)	13 (216)			0.37 (0.15, 0.89) *	0.027
Not all stools passed (yes/no)	13 (215)			0.62 (0.25, 1.52)	0.296
Constipation (yes/no) [d]	13 (216)			0.51 (0.22, 1.18)	0.116

[a] Incidence rate ratio (IRR) (= $mean_{probiotics} / mean_{no\ probiotics}$) from Poisson mixed-effects model for count symptoms. IRR<1 means that probiotics reduce symptoms.
[b] Odds ratios on increasing to the next higher level of symptom with probiotics, from ordinal logistic mixed-effects model for ordinal outcomes. The symptom is in a 5-point Likert scale, with 1=lowest and 5=highest. OR<1 means that probiotics reduce score.
[c] Participants without vomiting during enrollment were excluded in analysis.
[d] Constipation is defined as yes to any one of those questions listed below: Stools are hard, difficult to pass, or not all passed.

Table S3. Estimated ITT and PP effects of probiotics on bacteria and metabolites based on linear mixed-effects models adjusted by gestational age. To adjust for multiple comparisons, Benjamini-Hochberg procedure was used to adjust raw *p*-values for 123 metabolites, top 50 genus, and top 100 species, respectively. Confidence intervals were not adjusted by multiplicity. Only biomarkers with raw *p*-values<0.05 were reported. ** indicates *p* < 0.01, * indicates *p* < 0.05.

Population	Biomarkers	Number of Participants (Observation Days)	Fold Change [a] (95% CI)	Raw *p*-Value	Adjusted *p*-Value	Difference [b] (95% CI)	Raw *p*-Value	Adjusted *p*-Value
**ITT**	**Metabolites**							
	α-Tocopherol	11 (73)	1.40 (1.11, 1.76)	0.005 **	0.59			
	Fucose	11 (73)	1.38 (1.00, 1.89)	0.05 *	0.95			
	**Genus abundance (%)**							
	Akkermansia	11 (73)				-0.67 (-1.24, -0.10)	0.021 *	0.84
	**Species abundance (%)**							
	Akkermansia muciniphila	11 (73)				-0.61 (-1.16, -0.07)	0.027*	0.92
**PP (sensitivity analysis)**	**Metabolites**							
	α-Tocopherol	9 (60)	1.42 (1.09, 1.85)	0.01 **	0.61			
	Ribose	9 (60)	1.54 (1.00, 2.38)	0.05 *	0.99			
	Octadecanol	9 (60)	1.34 (1.12, 1.61)	0.002 **	0.26			
	Inosine	9 (60)	0.67 (0.46, 0.97)	0.04 *	0.99			
	**Genus abundance (%)**							
	Acetobacterium	9 (60)				-0.17 (-0.34, -0.01)	0.038 *	0.67
	Mordavella	9 (60)				0.07 (0.00, 0.13)	0.040 *	0.67
	Akkermansia	9 (60)				-0.73 (-1.42, -0.04)	0.039 *	0.67
	**Species abundance (%)**							
	Mordavella sp. Marseille-P3756	9 (60)				0.06 (0.00, 0.11)	0.034 *	0.89

	Akkermansia. muciniphila	9 (60)		-0.66 (-1.32, -0.00)	0.049 *	0.89
	Lachnospirace bacterium GAM79	9 (60)		-1.34 (-2.47, -0.20)	0.022 *	0.89

[a] Metabolites were log-transformed, and fold changes (exponentiated coefficients) due to probiotic intake were reported. Fold change >1 means that metabolites increase with probiotics.
[b] Difference (= $\text{mean}_{\text{probiotics}}$ - $\text{mean}_{\text{no probiotics}}$) from linear mixed-effects model. Difference<0 means that probiotics reduce abundance.

Table S4. Sensitivity analyses in PP population. Estimated effects of probiotics on fecal gene copy numbers, based on linear mixed-effects models adjusted by gestational age. ** indicates *p* < 0.01, * indicates *p* < 0.05.

Biomarkers	Number of Participants (Observation Days)	Fold Change [a] (95% CI)	*p*-Value
**Butyric acid-producing genes**			
bcoA	22 (134)	0.93 (0.72, 1.21)	0.61
buk	22 (134)	0.99 (0.71, 1.40)	0.98
**Bile acid-producing genes**			
bsh	22 (134)	6.10 (3.44,10.82)**	<0.001
baiJ	22 (134)	0.79 (0.46, 1.33)	0.36

[a] Gene copy numbers were log-transformed, and fold changes (exponentiated coefficients) due to probiotic intake were reported. Fold change>1 means that the gene copy number increases with probiotics.

Table S5. Association between biomarkers on Day 0 and symptoms in ITT and PP population, based on generalized linear mixed-effects models. Fixed effects included probiotics, gestational age, each biomarker at Day 0 and its interaction between probiotics. Interactions between biomarkers and probiotics were removed from final model due to no significance. ** indicates *p*<0.01, * indicates *p*<0.05. Confidence intervals and *p*-values were not adjusted by multiplicity.

Population	Symptoms	Number of Participants (Observation Days)	Incidence Rate Ratio [a] (95% CI)	*p*-val ue	Odds Ratio [b] (95% CI)	*p*-va lue
ITT	**Biomarker = log-transformed α-tocopherol level**					
	Daily nausea score (1-5)	11 (182)			0.28 (0.03, 2.42)	0.25
	Daily vomit score (1-5) [c]	4 (68)			0.20 (0.05, 0.76) *	0.02
	Daily nausea times	11 (182)	0.75 (0.27, 2.04)	0.57		
	Daily vomit times [d]	4 (68)	0.43 (0.17, 1.13)	0.09		
	**Biomarker = *Akkermansia* abundance (%)**					
	Daily nausea score (1-5)	11 (182)			0.74 (0.47, 1.17)	0.20
	Daily vomit score (1-5)[c]	4 (68)			1.70 (0.71, 4.03)	0.24
	Daily nausea times	11 (182)	0.90 (0.74, 1.11)	0.32		
	Daily vomit times[d]	4 (68)	1.85 (1.27, 2.70) **	0.002		
	**Biomarker = *A. muciniphila* abundance (%)**					
	Daily nausea score (1-5)	11 (182)			0.71 (0.42, 1.22)	0.22
	Daily vomit score (1-5) [c]	4 (68)			1.69 (0.71, 4.02)	0.24
	Daily nausea times	11 (182)	0.89 (0.70, 1.13)	0.34		
	Daily vomit times [d]	4 (68)	1.85 (1.27, 2.69) **	0.002		
PP (sensitivity analysis)	**Biomarker = log-transformed α-tocopherol level**					
	Daily nausea score (1-5)	9 (150)			0.12 (0.01, 1.36)	0.09
	Daily vomit score (1-5) [c]	4 (68)			0.20 (0.05, 0.76) *	0.02
	Daily nausea times	9 (150)	0.52 (0.17, 1.53)	0.24		
	Daily vomiting times[d]	4 (68)	0.43 (0.17, 1.13)	0.09		

	**Biomarkerr = *Akkermansia* abundance (%)**					
	Daily nausea score (1-5)	9 (150)			0.77 (0.47, 1.28)	0.31
	Daily vomit score (1-5) [c]	4 (68)			1.70 (0.71, 4.03)	0.24
	Daily nausea times	9 (150)	0.94 (0.76, 1.16)	0.56		
	Daily vomit times[d]	4 (68)	1.85 (1.27, 2.70) **	0.002		
	**Biomarker = *A. muciniphila* abundance (%)**					
	Daily nausea score (1-5)	9 (150)			0.75 (0.41, 1.35)	0.34
	Daily vomit score (1-5) [c]	4 (68)			1.69 (0.71, 4.02)	0.24
	Daily nausea times	9 (150)	0.93 (0.73, 1.20)	0.59		
	Daily vomit times [d]	4 (68)	1.85 (1.27, 2.69) **	0.002		

[a] Incidence rate ratio (IRR) of 1-unit increase in biomarker on Day 0, based on Poisson mixed-effects model for count outcomes. IRR<1 means that patients with high biomarker level on Day 0 are associated with a low outcome during the entire study (after adjusting for probiotic effect).
[b] Odds ratios on increasing to the next higher level of symptoms when there is a 1-unit increase in biomarker on Day 0, based on ordinal logistic mixed-effects model for ordinal outcomes. The outcome is in a 5-point Likert scale, with 1=lowest and 5=highest. OR<1 means that patients with a high biomarker level on Day 0 are associated with low symptom scores during the entire study (after adjusting for probiotic effect).
[c,d] Participants without vomiting during enrollment were excluded.

Table S6. Sensitivity analyses in PP population. Association between *bsh* copy numbers on Day 0and symptoms, based on generalized linear mixed-effects models. Fixed effects included probiotics, gestational age, *bsh* at Day 0 and its interaction between probiotics. Interaction between *bsh* and probiotics was removed from final model due to no significance. ** indicates *p* < 0.01, * indicates *p* < 0.05.

	**Biomarker = log-transformed *bsh* copy number**				
**Symptoms**	**Number of Participants (Observations) in Analysis**	**Incidence Rate Ratio [a] (95% CI)**	***p*-value**	**Odds Ratio [b] (95% CI)**	***p*-Value**
Daily nausea score (1-5)	22 (367)			0.74 (0.29, 1.85)	0.52
Daily vomit score (1-5) [c]	9 (152)			0.26 (0.06, 1.18)	0.08
Daily nausea times	22 (366)	0.95 (0.71, 1.26)	0.70		
Daily vomit times [d]	9 (152)	0.50 (0.28, 0.89) *	0.02		

[a] Incidence rate ratio (IRR) of 1-unit increase in log-transformed *bsh* level on Day 0, based on Poisson mixed-effects model for count outcomes. IRR<1 means that patients with high *bsh* copy numbers on Day 0 are associated with low outcomes during the entire study (after adjusting for probiotic effect).

[b] Odds ratios on increasing to the next higher level of symptoms when there is a 1-unit increase in log-transformed *bsh* on Day 0, based on ordinal logistic mixed-effects model for ordinal outcomes. The symptom is in a 5-point Likert scale, with 1=lowest and 5=highest. OR<1 means that patients with high bsh levels on Day 0 are associated with a low symptom score during the entire study (after adjusting for probiotic effect).

[c,d] Participants without vomiting during enrollment were excluded.

References:

1. Administration, F.a.D. Adjusting for Covariates in Randomized Clinical Trials for Drugs and Biologics with Continuous Outcomes Guidance for Industry. 2019.
2. Yoshimoto, S., T.M. Loo, K. Atarashi, et al. Obesity-induced gut microbial metabolite promotes liver cancer through senescence secretome. *Nature*, 2013. *499*(7456): p. 97-101.
3. Li, C.Y., J.L. Dempsey, D. Wang, et al. PBDEs Altered Gut Microbiome and Bile Acid Homeostasis in Male C57BL/6 Mice. *Drug Metab Dispos*, 2018. *46*(8): p. 1226-1240.
4. Louis, P. and H.J. Flint. Development of a semiquantitative degenerate real-time pcr-based assay for estimation of numbers of butyryl-coenzyme A (CoA) CoA transferase genes in complex bacterial samples. *Appl Environ Microbiol*, 2007. *73*(6): p. 2009-12.
5. Vital, M., C.R. Penton, Q. Wang, et al. A gene-targeted approach to investigate the intestinal butyrate-producing bacterialcommunity. *Microbiome*, 2013. *1*(1): p. 8.
